# Building a scalable diabetic limb preservation program: four steps to success

**DOI:** 10.1080/2000625X.2018.1452513

**Published:** 2018-03-22

**Authors:** Tanzim Khan, Laura Shin, Stephanie Woelfel, Vincent Rowe, Brittany L. Wilson, David G. Armstrong

**Affiliations:** a The Southwestern Academic Limb Salvage Alliance (SALSA), Department of Surgery, Keck School of Medicine of University of Southern California (USC), Los Angeles, CA, USA; b Department of Orthopaedics, University of Pittsburgh Medical Center (UPMC), Pittsburgh, PA, USA; c Division of Biokinesiology and Physical Therapy, University of Southern California (USC), Los Angeles, CA, USA

**Keywords:** Diabetic foot, ulcer, amputation, diabetic foot remission, recurrence, screening, clinics

## Abstract

Over the past generation, limb preservation programs and diabetic foot services have begun to proliferate within academic health science centers as well as within health-care systems in general. We describe four key components for a successful program that, developed sequentially with temporal overlap, can allow the program to scale. The first component includes establishment of a ‘hot foot line’ for urgent emergency department/inpatient referral. The second includes development of a wound-healing clinic to address outpatient care through to remission. The third component focuses on the diabetic foot in remission to maximize ulcer-free days following healing. The fourth and final component focuses on implementation of local and widespread screening clinics to identify and triage patients into appropriate therapeutic and surveillance programs for healing, remission, and primary prevention. Along with developing each of these components, we describe discrete methods to quantify success.

## Introduction

The population of people affected by diabetes is rising. It has increased by nearly 400% in the last generation within the USA alone [1]. It is estimated that up to one third of people diagnosed with diabetes will develop a foot ulcer [2]. Non-healing or chronic ulcerations can lead to infection and subsequent amputation [3]. Five-year mortality of people with wounds, peripheral artery disease, and/or amputation exceed the most aggressive cancers [1,4].

To combat the increasing complications of the diabetic foot and resulting morbidity, limb preservation programs have begun to proliferate. Limb preservation programs consists of interdisciplinary teams that work synergistically to exchange information, discuss assessment, and create joint plans to achieve the goal of limb salvage in an integrated model. This model often incorporates podiatric and vascular surgeons (‘Toe and Flow’ model) as the core of an interdisciplinary team but which may include a diverse group of health professionals [5–8]. We have previously identified seven essential skills, first described by Fitzgerald and co-workers and later refined to eight skills by Wukich and colleagues, to define the key skill sets associated with care across a limb preservation program (Table 1) [9,10]. The partnership of these specialists allows for rapid coordinated management of vascular, mechanical, and soft tissue reconstruction.

**Table 1.** Eight collective clinical skills for members of a limb preservation program.

1. The ability to perform hemodynamic and anatomic vascular assessment with revascularization, as necessary.
2. The ability to perform neurologic workup.
3. The ability to perform site-appropriate culture technique.
4. The ability to perform wound assessment and staging/grading of infection and ischemia.
5. The ability to perform site-specific bedside and intraoperative incision and debridement.
6. The ability to initiate and modify culture-specific and patient-appropriate antibiotic therapy.
7. The ability to perform appropriate postoperative monitoring to reduce risks of re-ulceration and infection [5,9,10].
8. The ability to provide basic foot care education and referral into (and monitoring of) a home education program [3,11,10].

The assembly of a motivated interdisciplinary team is an essential initial element to establishing a limb salvage team and successful limb preservation program. There are four additional components which we and others have found to be important in creating a truly comprehensive local, regional, or national initiative. These components include initiating a ‘hot foot line’, having access to or establishing a wound-healing clinic, a remission clinic, and a screening clinic. In addition to these four components, a limb preservation program should monitor measurable outcomes to assess and demonstrate the validity, efficacy, and long-term outcomes of these specialized programs. The purpose of this manuscript is to outline these four components and measurable outcomes.

## Step one: establishment of a ‘hot foot line’

### Acute inpatient care for acute-on-chronic complications

In the acute setting, diabetic foot complications presenting to the emergency room or on the inpatient wards should be reported to a single ‘hot foot line’ [12]. Once engaged, the limb salvage team will rapidly assess the patient and internally triage the patient. Key internal questions that will determine the primary management team within the limb preservation team are whether the problem involves infection, ischemia, or a combination of both infection and ischemia [13].

For example, if the patient presents with tissue loss, infection, and palpable pulses, the podiatric surgeon will be the primary consult to address the lower extremity wound and infection [14]. The podiatric surgical team may internally consult with vascular surgery if there are signs of ischemia or if the foot is unsalvageable and a major primary amputation is needed.

If a patient presents with non-palpable pulses and a concomitant infection is present, vascular surgery will be the primary managing surgeon for the treatment of ischemia and infection. The vascular team may internally consult with podiatric surgery for initial debridement and infection control in an effort to preserve the limb. After the nidus of infection is removed or concomitant with the debridement, the vascular members of the team will perform revascularization.

If a patient is to present with ischemia without signs of acute infection, the initial primary consultant within the team would be the vascular surgeon. If revascularization is performed by the vascular surgeon with established arterial outflow to the extremities, the podiatric surgeon may be consulted to perform appropriate reconstructive surgery or partial-foot amputations as needed [5,9,10]. Once the acute infection and/or vascular reconstruction is addressed, the patient may now move into an outpatient setting, as their active condition is no longer complicated by infective or ischemic processes, and management is now geared toward tissue loss.

## Step two: development of wound-healing clinics

### Outpatient care to manage tissue loss

A wound clinic should be established to evaluate and treat active tissue loss. The primary focus of the clinic is to determine the etiology of the lower extremity wound and employ appropriate modalities to rapidly accelerate wound closure, moving patients into remission [2]. Neuropathic wounds require biomechanical analysis and proper off-loading management. Ischemic wounds require vascular diagnostics and potential surgical intervention. Neuro-ischemic wounds require a combination of both aforementioned treatments. The wound-healing clinic should stock the appropriate tools and supplies to treat lower extremity wounds which include debridement instruments, total contact casts or their equivalents, dressings for various wound beds, and have access to radiographs and noninvasive vascular testing.

Ideally, this should be an interdisciplinary clinic that includes specialists trained in the reconstruction of the foot and ankle, as well as those trained in vascular reconstruction. It is preferable to have both specialists available concurrently for the evaluation of complex cases where the expertise of both specialties is needed for planning and intervention. If this is not feasible, then a close-knit working relationship is required of the two specialists to synergistically improve patient outcomes [8]. Physical therapy-centric wound-healing clinics nested within this model can also be very useful as they provide highly skilled services that may allow for active day-to-day operations [15,16].

## Step three: remission clinic

### Outpatient care to extend ulcer-free, hospital-free, and activity-rich days

After resolution of sequelae from wounds and complete healing is obtained, the patient should be referred to a remission clinic [17,18]. Regardless if the patient was managed as an inpatient or an outpatient, the remission clinic serves as an integral part of a limb preservation program to maximize ulcer-free, hospital-free, and activity-rich days [2,18]. Individualized patient self-care and monitoring education is a key function of the remission clinic, along with home-based monitoring program coordination, as they have shown to reduce ulcer recurrence [2,19–22]. A gradual return to activity is important in extending remission. The remission clinic offers a comprehensive approach to safely achieve this goal. The elements of magnitude, loading time, and direction of tissue stress need to be managed in an active patient to prevent re-injury. Consultation with a physical therapist can provide an exercise prescription to achieve this goal [23]. Biomechanical evaluation of the patient’s extremities may indicate the need for either external accommodation (shoe gear, prostheses, and insoles) or reconstructive surgery. Reconstructive surgery and partial foot amputations can cause a shift in the complex biomechanics of the foot and ankle. These patients need to be evaluated for high pressure points to prevent areas of new injury or re-injury. Collaboration with a pedorthist or prosthetist to construct accommodative devices that off-load areas of high pressure and help distribute pressure more evenly can be advantageous, especially in the plantar foot. However, many of these devices can alter the patient’s gait mechanics. In that scenario, a comprehensive movement analysis can assist in maximizing patient safety, mitigate fall risk, and determine whether an assistive device may be beneficial. Management of at-risk tissue, such as hypertrophic tissue formation at sites of increased plantar pressure in the neuropathic patient, is also an integral responsibility of the remission clinic. This might be accomplished via tissue debridement and off-loading, or by reconstructive surgery for recalcitrant areas [24].

## Step four: screening clinics

The final component of a comprehensive limb preservation program is development and dissemination of screening services. Ideally, every person with diabetes will be screened at least annually for risk factors for lower extremity complications. This would include a detailed examination of the lower extremity in regard to the cardiovascular, neurological, dermatological, and musculoskeletal systems. This examination has been codified as part of the American Diabetes Association Comprehensive Diabetic Foot Examination [25]. It was further pared down to a basic ‘three-minute foot exam’ for family physicians, nurses, and technicians [11,26–28]. Once risk levels are determined, more appropriate recommendations can be made regarding shoe-gear, insoles, orthoses, and follow-up intervals. From within this clinic, referrals to the appropriate specialists can be made and care can be escalated as needed to the remission clinics (for those with history of pathology), wound-healing clinic (for active non-limb threatening injuries), or the ‘hot foot line’ (when limb threatening pathology is present). Beyond this, community clinics and in-hospital education programs may be helpful in identifying patients of all risk strata and enrolling them into the treatment or surveillance program from which they may benefit [29].

## Auditing

**Figure 1.**
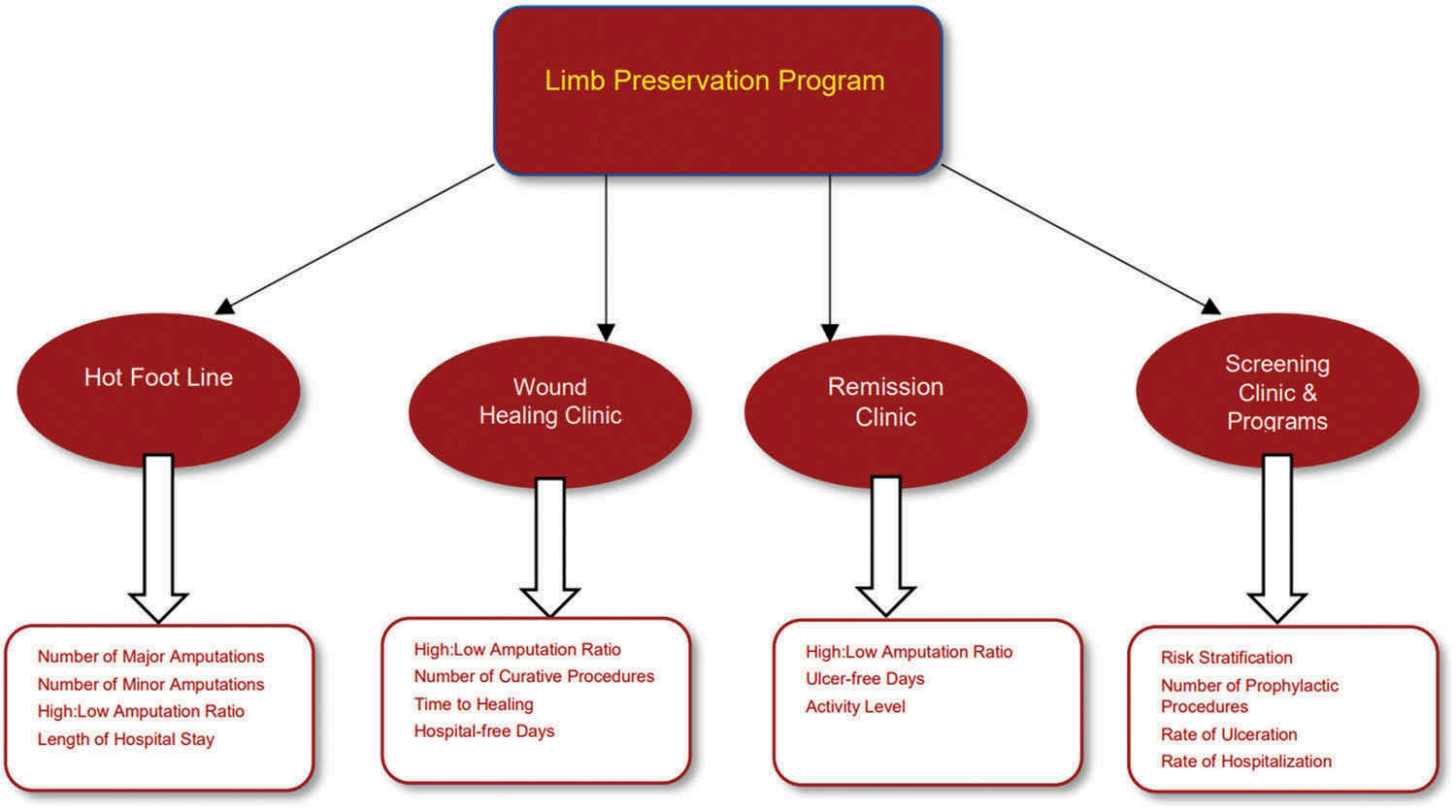
Structure and measurable outcomes for each component of a limb preservation program.

The implementation of continuous prospective data collection is essential to the limb preservation program [30]. The data collected utilizing measurable outcomes is a key component in quality improvement and monitoring patient progress. Every component of the limb preservation program can be evaluated separately or as a whole unit (Figure 1). For the ‘hot foot line’, one can track the in-hospital and regional rate of amputation. These data can be further delineated to study the ratio of major to minor amputations (High:Low amputation ratio) [31]. A third component to audit is the length of stay in the hospital per event. In the wound-healing clinic, one can consider measuring the High:Low amputation ratio, the time to healing, the number of curative procedures, and the rate of hospitalization. The remission clinic data might include ulcer-free days, functional activity level, as well as previously mentioned data points such as high-low amputation ratio. The screening clinics can provide prospective data on long-term outcomes after the initial risk is assessed. Additional data collection and analysis for the screening clinic are similar to those mentioned for the remission clinic.

## Conclusion

A successful limb preservation program begins and ends with an enthusiastic, cohesive, interdisciplinary team. With this foundation in place, the four steps to building a scalable single site, regional or national limb preservation program should include (in order): implementation of a hot foot line, wound-healing clinic, remission clinic, and screening clinics. The measured outcomes from each of these components will contribute to an audit that will ensure quality improvement through assessment and review, as well as establish benchmarks for quality of care.
